# Post more! The mediating role of social capital between Instagram use and satisfaction with life

**DOI:** 10.1007/s12144-021-02579-6

**Published:** 2021-12-03

**Authors:** Linda-Elisabeth Reimann, Phillip Ozimek, Elke Rohmann, Hans-Werner Bierhoff

**Affiliations:** 1 Department of Psychology, University of Hagen, Hagen, Germany; 2 Department of Social Psychology, Ruhr University of Bochum, Bochum, Germany

**Keywords:** Instagram use, Social media, Social capital, Satisfaction with life, Well-being

## Abstract

Since more and more people have begun to use social networking sites (SNSs), research on the use of SNSs is flourishing. This study examines Instagram use and the psychological well-being of the users. It was conducted based on two samples (*n*_1_ = 143 and *n*_2_ = 320) examining the relationship between Instagram use, social capital, and satisfaction with life using online questionnaires. Social capital was divided into bonding and bridging social capital and Instagram use was distinguished depending on an active and passive mode, respectively. Instagram use was measured by a behavioral report – the Instagram Activity Questionnaire (IAQ) – which was developed in accordance with the Facebook-Activity Questionnaire (FAQ; cf., Ozimek & Bierhoff, 2016). The results indicated consistently in both samples the occurrence of positive associations between mode of Instagram use and social capital variables. Furthermore, only bonding social capital – not bridging social capital – was positively correlated to satisfaction with life. A path model showed that the negative association of active Instagram use and satisfaction with life was positively mediated by bonding social capital. These results are discussed based on social capital theory. Limitations of this investigation are pointed out and suggestions for future research are outlined.

## Introduction

Over the last decade, the use of *Social Networking Sites* (SNSs), such as Facebook, Instagram, or Snapchat have become a daily routine for more and more people Therefore, their popularity increased rapidly (Pew Research Center, 2021). SNSs represent Internet platforms which enable communication in social relationships. Research on SNSs includes Instagram (Highfield & Leaver, 2015; Sherlock & Wagstaff, 2019), which provides features like interacting with other users, posting pictures, following sites of interest, and using hashtags. Instagram users can follow other users – so-called *followees* – such as friends, famous people or sites posting content users are interested in (i.e., cooking, fitness, comedy, latest news etc.). By following others, users can see new posts or stories (pictures or videos) that have been shared recently and are also able to like and/or comment the content. They become so-called *followers*. Other features Instagram provides are the chat function, story-highlights, the explore-function or the shop, where users can directly buy products, they may like. Prior investigations show that Instagram had the highest influence on its users compared to other SNSs (cf., Krallman et al., 2016) and became a leading SNS in the last decade (Ting, 2015). Furthermore, Waterloo et al. (2018) revealed that positive emotions were mostly expressed on WhatsApp and Instagram, whereas Instagram was on the last place in terms of expression of negative emotions.

In general, we focus on two study aims: First, to implement a new measurement instrument as a reliable and valid measure for Instagram activity. Second, to investigate how Instagram use relates to social capital and satisfaction with life.

## Theoretical Background

### Satisfaction with Life

Satisfaction with life “refers to a cognitive, judgmental process” (Diener et al., 1985, p. 71) in which people compare their own life situation with the expected standard of other people. It is a well-established and valid construct in social and positive psychology which represents the basis for subjective well-being (Pavot & Diener, 2008). Research showed associations to many other individual-differences variables such as emotional intelligence and job satisfaction (Ignat & Clipa, 2012), self-esteem (Hong & Giannakopoulos, 1994), and perceived stress (Samaha & Hawi, 2016). Clearly, these concomitants of satisfaction with life refer to central domains of human life.

There are many studies focusing on the relationship between SNSs and satisfaction with life (cf., Brailovskaia & Margraf, 2016, 2018; Ellison et al., 2007; Fioravanti et al., 2020; Kross et al., 2013; Manago et al., 2012). However, their results were partially contradictory and different measurements were utilised to investigate satisfaction with life (e.g., the Student’s Life Satisfaction Scale in the work of Manago et al., 2012). Most of past research used the Satisfaction with Life Scale by Diener et al. (1985). Recent studies explored whether there are differences on mental-health variables, such as satisfaction with life, between Facebook users and non-users. Results showed that Facebook users scored higher on mental-health variables than non-users (Brailovskaia & Margraf, 2016; Ljepava et al., 2013). Another study by Brailovskaia and Margraf (2018) revealed a positive correlation between the use of Facebook as well as Instagram and satisfaction with life, whereas Twitter use was negatively correlated with satisfaction with life.

However, other studies found opposite effects. Kross et al. (2013) reported evidence for a negative relationship between intensive Facebook use and satisfaction with life in a longitudinal investigation. In an experimental investigation Tromholt (2016) demonstrated that cognitive and affective well-being was higher after participants quitted Facebook use for one week. A similar result was found in an experimental investigation (Allcott et al., 2020) in which Facebook users scored higher on subjective well-being after four weeks without using Facebook. Though, the same work also indicated that Facebook “provides large benefits for its users” (Allcott et al., 2020, p.36) due to group and social life activities or the provision of opportunities to receive news and information. Another recent study found that quitting Instagram for one week resulted in higher levels of satisfaction with life (Fioravanti et al., 2020). In addition, Facebook use may foster media addiction which in turn is associated positively with negative well-being including insomnia (Brailovskaia et al., 2019).

In summary, past research revealed that the way Facebook, Instagram or SNSs in general affect satisfaction with life is still unsettled. More research is needed to better understand why past results have been contradictory. However, these contradictory results are not surprising because research has also shown that SNSs are being used quite differently (cf., Gazit et al., 2019; Hayes et al., 2015). Verduyn et al. (2015) provided experimental evidence on the different consequences of active vs. passive SNS use. They showed that well-being of participants decreased after ten minutes of using Facebook passively, whereas well-being increased after active Facebook use.

Facebook studies have also ascertained relevant additional findings explaining these differences. Kim and Lee (2011) found that positive self-presentation had a direct positive effect on subjective well-being, whereas honest self-presentation had an indirect positive effect on subjective well-being via perceived social support. In addition, another study revealed that larger Facebook networks (meaning more comments and likes from Facebook friends compared with smaller networks) predicted higher levels of satisfaction with life (Manago et al., 2012). Thus, it can be suggested that, regardless of active or passive SNS use, social capital has an independent effect on satisfaction with life. This leads to the conclusion that the effect Instagram use has on satisfaction with life could be influenced by perceived social capital (cf., Brailovskaia & Margraf, 2018; Ellison et al., 2007). This possibility is considered next.

### Social Capital

Social capital refers to the resources people receive from the relationships they have. It can be divided into two different types: *bridging* and *bonding* social capital (Putnam, 2000). Bridging social capital applies to distant relationships and loose connections to other people which might offer possibilities such as getting a work placement or participating in cooperative endeavours. Bonding social capital refers to close relationships within the in-group including emotional support, such as family and close friends (Morrow, 2001).

Wellman et al. (2001, p. 450) inferred “that the Internet is particularly useful for keeping contact among friends who are socially and geographically dispersed” and for relationship maintenance in general (Tong & Walther, 2011). The benefits of SNSs, with respect to the accumulation of social capital, have been studied intensively. Social relationships provide different resources for the persons involved which fit into the framework of social capital (Ellison & Vitak, 2015). Depending on the kind of resources mobilized bridging and bonding social capital are distinguished. Whereas bridging social capital is provided by access to the broader social community (based on “weak ties”), bonding social capital is primarily derived from the social network of friends and family members (based on “strong ties”). The use of SNSs may create a sense of relatedness and, therefore, provides sources of bridging and bonding social capital. Prior investigations, which were summarised by Ellison and Vitak (2015), have revealed associations between the use of different SNSs on the one hand and social capital gain on the other hand. The benefits of SNS use include question-and-answer exchange, getting social support and emotional exchange. These benefits are frequently obtained on the basis of generalized reciprocity (in contrast to specific reciprocity), i.e., reciprocity which goes beyond one specific relationship. Generalized reciprocity means that a favour received in one relationship is paid back by a favour given in another relationship (Putnam, 2000). In addition, the number of *actual* friends is a better indicator of the users’ social capital derived from SNSs than the *total* number of friends in the network indicating that quality of social contacts is more important than quantity (Ellison et al., 2011).

Results indicated that Facebook use has a stronger impact on bridging than on bonding social capital (Ellison et al., 2007). Furthermore, Facebook users exhibiting low satisfaction with life and low self-esteem benefited more from intense Facebook use, which could also be confirmed in a longitudinal study (cf., Steinfield et al., 2008). Further results revealed that bridging social capital achieved the highest level for the use of Twitter, whereas bonding social capital achieved the highest level for the use of Snapchat (Phua et al., 2017). Instagram users reported slightly higher levels for bridging than for bonding social capital. The authors argued that this result “may be attributed to Twitter being a micro-blogging platform” (Phua et al., 2017, p. 13). In contrast, on Facebook and Snapchat users mostly interact with friends from real life. Furthermore, Instagram seems to be a combination of both because “users are also likely to interact with others they do not know in real life, but to a lesser degree than Twitter” (Phua et al., 2017, p.13).

With respect to mode of SNS use, prior research indicated that activities on SNSs can be divided into *active* and *passive* modes of usage (cf., Burke et al., 2010; Krasnova et al., 2013; Verdyn et al., 2015). The distinction between active and passive modes of SNS usage is important because different effects on well-being are likely to emerge. Active SNS use generally represents a conscious interaction in the sense of social content generation (e.g., creating a story or commenting on others’ contributions), whereas passive SNS use means consuming user-generated social content (Wang et al., 2018). Hence, social exchange within active use describes an (inter)active process. Passive use refers to a one-way and solely receiving activity (Meier & Reinecke, 2020).

More specifically, studies showed, on the one hand, that passive SNS use is negatively associated to well-being (cf., Burke et al., 2013; Krasnova et al., 2013; Verduyn et al., 2015). On the other hand, active use was positively associated to well-being (cf., Chou & Edge, 2012; Deters & Mehl, 2013; Ellison et al., 2007). The difference between the consequences of active and passive use of SNSs can possibly be clarified by variables mediating the association between SNS use and well-being. In this context, Sagioglou and Greitemeyer (2014) found that the decrease of well-being after passive SNS use was mediated by the feeling of having wasted time. In contrast, the increase of well-being after active use was mediated by the development of social capital (cf., Burke et al., 2010; Ellison et al., 2007).

SNSs enable the maintenance of a broad spectrum of social relationships including social support, information exchange, and chatting (Ellison & Vitak, 2015), which is summarized as social capital in the present study. Previous research indicated that the accumulation of social capital via social relationships constitutes the most important determinant of satisfaction with life (Berscheid & Reis, 1998). Based on this result which refers to offline relationships it can be suggested by generalization to online relationships that Instagram use is in general correlated with mobilization of social capital (*H1*) and satisfaction with life (*H2*). *H1* includes that, first, active Instagram use is positively correlated with bonding social capital (*H1a*). Second, passive Instagram use is positively correlated with bonding social capital (*H1b*). Third, active Instagram use is positively correlated with bridging social capital (*H1c*), and fourth, passive Instagram use is positively correlated with bridging social capital (*H1d*).

Although Ellison et al. (2007) indicated that bridging social capital had the strongest associations with SNSs use, bonding social capital had the strongest association with satisfaction with life (Yeo & Lee, 2019). It has to be taken into account that the study by Ellison and her colleagues was conducted almost 15 years ago and the way people use SNSs has changed since then. In the past, SNSs were an instrument to meet new people and stay in contact with old friends or schoolmates. This activity was characterised as based on weak ties belonging to bridging social capital (Putnam, 2000). However, as mentioned before, SNSs occupy a huge part of daily social life of their users. Currently it is a widespread habit to interact with a lot of people on SNSs and to maintain even very close friendships that mobilize bonding social capital (Shane-Simpson et al., 2018). Therefore, especially the domain of bonding social capital could influence the relationship of Instagram use and satisfaction with life. In addition, studies show that active users of SNSs benefited more from social capital and that Instagram users following fewer strangers exhibited positive associations with depressive symptoms (Burke et al., 2010; Lup et al., 2015). In addition, Waterloo et al. (2018) revealed that positive emotions were mostly expressed on WhatsApp and Instagram.

In accordance with the differences between active and passive SNS use (cf., Chou & Edge, 2012; Burke et al., 2013; Ellison et al., 2007; Deters & Mehl, 2013; Krasnova et al., 2013; Verdyn et al., 2015) it can be assumed that active Instagram use is positively correlated with satisfaction with life *(H2a),* whereas passive Instagram use is negatively correlated with satisfaction with life (*H2b*). These hypotheses were derived in an effort to overcome inconsistent results regarding the association of satisfaction with life and SNSs. Especially the study by Verduyn et al. (2015) provided evidence that these inconsistent results can be resolved by taking the distinction between active and passive SNS use into account.

Based on theoretical and empirical considerations it can be suggested that perceived bonding social capital on Instagram is positively correlated with satisfaction with life *(H3)* because people constantly see content from close friends or interact with them on Instagram. Therefore, this hypothesis is based on strong-ties social networks as the basis of bonding social capital which facilitate the development of trust and reciprocity. Furthermore, elaborating the link between bonding social capital and satisfaction with life (cf., Burke et al., 2010; Ellison et al., 2007) it can also be hypothesised that gains of bonding social capital mediate the relationship between active Instagram use and satisfaction with life *(H4).* According to this, people who experience more bonding social capital on Instagram, due to an active interaction with close friends, would report higher values of satisfaction with life (active Instagram use → bonding social capital → satisfaction with life). This hypothesis goes beyond H3 by referring to a possible mediator connecting active Instagram use and satisfaction with life indirectly.

Nevertheless, other interpretations of the underlying framework are also possible. Specifically, the confirmation of each hypothesis would also be congruent with a revised *H4* which postulates that satisfaction with life influences active Instagram use via bonding social capital (satisfaction with life → bonding social capital → active Instagram use). In general, because we base our hypotheses on correlational data, no causal inference from the results are viable. But the revised sequence of influence is less plausible than the sequence of influence specified in *H4.* First, prior research indicated that social relationships represent the basis of satisfaction with life and not vice versa (for a summary of this research see Berscheid & Reis, 1998). In addition, bonding social capital was mobilized by in-group relationships which follow from active or passive Instagram use (Ellison & Vitak, 2015). In contrast, it is less plausible to assume that bonding social capital follows from satisfaction with life. Furthermore, bonding social capital is unlikely to instigate active Instagram use. Social capital theory assumes that social relationships facilitate the accumulation of bonding (and bridging) social capital and not vice versa. In summary, empirical research and the theoretical framework of social capital correspond very well with *H4* but is less congruent with the alternative sequence running from bonding social capital to active Instagram use.

## Method

### Participants

Before data was collected the program G*Power (version 3.1.9.2) was employed to calculate how many participants would be needed for a sufficient sample (Faul et al., 2007, 2009). An apriori analysis (Linear multiple regression: Fixed model, *R*^2^ increase) was conducted and an effect size of 0.15 was assumed. The appropriate sample size turned out to be *n* = 107. We collected two samples to test our hypotheses. Each sample fulfilled the sample size criterion.

#### Sample 1

The first sample consisted of 143 participants (72 males, 122 females) who were Instagram users. Their mean age was 23.92 (*SD* = 4.84). 83.20% of the participants were students and most of them studied psychology (41.33%).

#### Sample 2

Sample 2 included 349 participants, but 29 participants were excluded because the control items were answered incorrectly, or the data set was incomplete. The final sample consisted of 320 participants with 66 males and 254 females. The mean age was 24.05 (*SD* = 5.86). Again, the majority of the participants were students (90.60%) with most of them psychology students (53.40%). Sample 2 only contained participants who already had an Instagram account. Data about the duration of overall and daily Instagram use was collected. 87.20% of the participants used Instagram daily. The duration of Instagram use varied from one to ten minutes per day (10.60%) to more than three hours (1.60%), but most participants used Instagram ten to thirty minutes per day (33.40%).

We deposit all used data sets at: https://osf.io/yw298/?view_only=8a287c68db1d4cfcb7973fca456b3a2f.

### Procedure

For both samples an online survey was constructed either via the platform Unipark (https://www.unipark.de) for sample 1 or via Qualtrics (https://www.qualtrics.com) for sample 2. Data collection of the first sample took place from March 2019 to July 2019, whereas data for the second sample was collected from February 2020 to April 2020. The recruitment of participants was the same for both samples following a snowball-sampling-technique. Specifically, campus wide e-mails and flyers were spread, the platforms “SurveyCircle” (https://www.surveycircle.com) and “PollPool” (https://www.poll-pool.com) were utilised, and links of the surveys were shared via social media sites (Facebook, Instagram, WhatsApp, and LinkedIn). Demographic variables, Instagram activity, social capital, satisfaction with life, and additional explorative variables were obtained.

### Measures

The following questionnaires were employed in both samples with the exception of the Facebook Activity Questionnaire (FAQ), which was only employed in sample 1.

#### Facebook-Activity Questionnaire (FAQ)

For assessing the amount of Facebook use, the FAQ created by McAndrew and Jeong (2012) was used, which had been validated with German-speaking respondents (Ozimek & Bierhoff, 2016). The thirty items of the scale refer to three domains of Facebook use: *Watching* (11 items; e.g., “I’m looking at other’s relationship status”), *Acting* (13 items; e.g., “I’m posting photographs”), and *Impressing* (6 items; e.g., “I’m struggling to decide which profile picture I would like to post”). A five-point Likert scale assessed Facebook activity, from 1 = “never” to 5 = “very often”, with higher scores indicating higher Facebook activity. The results of the study of Ozimek and Bierhoff (2016) indicated good internal consistencies for all three dimensions (αwatching = 0.83, αimpressing = 0.79, αacting = 0.77) and also exhibited good construct validity. The current investigation revealed relatively high levels of overall Facebook activity, i.e., *M* = 2.80, *SD* = 0.76 and good internal consistencies with αwatching = 0.86, αimpressing = 0.84, αacting = 0.78.

#### Instagram Activity Questionnaire (IAQ)

Based on the German version of the FAQ by Ozimek and Bierhoff (2016) and the Xing-Activity questionnaire by Brandenberg et al. (2019) a questionnaire for Instagram use was developed. Items were answered on a five-point Likert scale from 1 = “never” to 5 = “very often”. Higher scores represent higher assessment of the frequency of Instagram-activities. Specifically, 38 items were employed which are based on specific functions that are provided on Instagram. Data of sample 1 and 2 (*N* = 463) was pooled for the following exploratory factor analysis (EFA) of the IAQ. The Bartlett’s test (*X*^2^(703) = 8179.41, *p* < .001) and the Kaiser-Meyer-Olkin Measure of Sampling Adequacy (*KMO* = .906) indicated that the variables were suitable for factor analysis. According to the Kaiser-Guttman criterion, only factors with eigenvalues greater than one were included (Guttman, 1960; Kaiser, 1954). 30 items displayed distinct factor loadings on one of the two factors. Based on this as well as on theoretical considerations by Verduyn et al. (2015), a two-factor solution was chosen explaining 35.92% of variance in Instagram use. Therefore, the EFA resulted in 30 IAQ-items distinguishing between active and passive domains of Instagram use including 19 and 11 items, respectively. Sample items are: *active* “I plan specifically when I post a picture” and *passive* “I look at stories of other users”.

Furthermore, because it is not recommendable to conduct exploratory as well as confirmatory factor analyses (CFA) within the same data set (see Kline, 2015) we reanalyzed unpublished data from our own lab to check for construct validity. The used data can be reviewed at https://osf.io/yw298/?view_only=8a287c68db1d4cfcb7973fca456b3a2f. The sample was formerly used in a bachelor thesis at the Ruhr University Bochum and consisted of 177 participants with 35 males and 141 females and a mean age of 24.47 (*SD* = 7.08). 76.27% of the participants were students and 35.03% studied psychology. We conducted a CFA using MPlus 8.6 (Muthén & Muthén, 2017). We employed the mean and variance adjusted unweighted least squares method (ULSMV). Note that the ULSMV is a robust estimator with respect to model violations (Kline, 2005). We assessed the model fit by four statistics, including (a) the chi-square test statistic to test the hypothesis that the proposed model provides a plausible structure which can be found in the data (note that when sample sizes are large a significant chi-square is very likely; Bentler & Bonett, 1980), (b) the Comparative Fit Index (CFI; an acceptable fit is inferred if the CFI is 0.90 or higher), (c) the Tucker–Lewis index (TLI; an acceptable fit is inferred if the TLI is 0.90 or higher and (d) the Root Mean Square Error of Approximation (RMSEA; an acceptable fit is inferred if the RMSEA is equal to 0.08 or smaller). The CFA revealed an acceptable fit: chi-square, p < .05, CFI = .99, TLI = .99, RMSEA = .08.

For more information on the exact formulation of the items and the factor loadings see Appendix Table 3. In both studies, participants exhibited moderately high ratings of Instagram activity, i.e., *M*active = 2.26–2.68, *SD*active = 0.65–0.67; *M*passive = 3.32–3.54, *SD*passive = 0.57–0.71, with highest ratings for passive Instagram use. Reliability analyses indicate good internal consistencies for both domains (αactive = .87–.89; αpassive = .79–.85).

Furthermore, split-half reliability coefficients were assessed within the two subscales of the IAQ, for both samples separated. For sample 1, the reliability coefficient between the two halves of the scale with Spearman-Brown correction was for active use *r* = .75 and for passive use *r* = .80, respectively, and for sample 2 the coefficients were *r* = .81 for active use and *r* = .71 for passive use, which represent sufficiently high correlations confirming the good reliability of the IAQ (Moosbrugger & Kelava, 2012, p. 331).

We obtained additionally – only in sample 2 - the amount of time users spend on Instagram. In correspondence with expectations, this measure was significantly positively correlated with both domains of Instagram use: active *r*(318) = .34, *p* < .001 and passive *r*(318) = .40, *p* < .001.

Several analyses were carried out to confirm the validity of the Instagram-Activity Scale. For sample 1, the IAQ was correlated with the three domains of the FAQ (Ozimek & Bierhoff, 2016): *Watching*, *Impressing*, and *Acting*. Results indicate convergent validity because highly significant correlations between the three domains of Facebook use and both scales of Instagram use emerged (Appendix Table 4). The domains of active Instagram use and acting Facebook use reveal the highest correlation coefficient, *r*(140) = .45, *p* < .001.

#### Social Capital

For assessing Internet specific social capital, a questionnaire developed by Williams (2006) was used which measures two domains of social capital: *bonding* (e.g., “There are several people online/offline I trust to help solve my problems”) and *bridging* social capital (e.g., “Interacting with people online/offline makes me feel like a part of a larger community”). The word “Instagram” replaced “online/offline” to make the items even more specific. Another subscale of social capital is the so called *maintained* social capital that is included in the 14-item version of Leiner et al. (2010) who constructed a German version of the questionnaire. They reported internal consistencies of the questionnaire which ranged from α = .77 to α = .85. However, in the current investigation only the bonding and bridging domains of social capital were used, because how people use SNSs has changed over the last decade. Maintaining old relationships from the past may not be an important part of using SNSs anymore. The items were answered on a five-point Likert scale (from 1 = “strongly disagree” to 5 = “strongly agree”). Higher scores on this scale indicate higher levels of perceived social capital. In the two samples of this investigation participants reported relatively high levels for all three subscales of social capital, i.e., *M*bonding = 3.06–3.09, *SD*bonding = 1.10–1.12; *M*bridging = 3.12–3.25, *SD*bridging = 0.78–0.87 with highest values for bridging social capital. Reliability analyses showed satisfactory internal consistency coefficients, αbonding = .89–.90; αbridging = .75–.81, and with α = .85–.88 for both scales.

#### Satisfaction with Life

The satisfaction with one’s own life was measured with the Satisfaction with Life Scale (SWLS, Diener et al., 1985), which was validated in German by Glaesmer et al. (2011). The SWLS was chosen for this study because most of past research utilised this measurement for satisfaction with life for its high reliability and validity. “It has shown itself to be useful in a wide range of research settings and applications “(Pavot & Diener, 2008, p.148). The scale consists of five items (e.g., “in most ways my life is close to my ideal”) using a seven-point Likert response scale (from 1 = “I do not agree” to 7 = “I fully agree”). Internal consistencies ranged from α = .61 to α = .84 (Glaesmer et al., 2011). In the current study, participants reported modest to high levels of satisfaction with life, i.e., *M* = 5.16–5.20, *SD* = 1.11–1.19. Reliability analyses indicated good internal consistency coefficients of the SWLS, α = .87–.88.

### Statistical Analyses

For analysis of the data IBM SPSS 26 was used. The hypotheses were examined by conducting correlational analysis and mediation analysis. Furthermore, a test of replicability of the mediation analysis was conducted using the p-checker app (https://shinyapps.org/apps/p-checker/; accessed on 20 Mai 2021) in order to check whether the test has the quality of exact reproduction.

## Results

### Comparison between Samples

Additional *Χ*^2^-analyses were performed to compare whether both samples differ on gender and proportion of students. In addition, *T*-tests were used to compare the samples on age and the questionnaire measures of Instagram use, social capital, and satisfaction with life. The results are depicted in Table 1. The analyses indicated that the samples did not differ significantly depending on gender, *Χ*^2^(1) = 2.51, *p* = 0.113, but they differed significantly on proportion of students, *Χ*^2^(1) = 5.27, *p* = 0.022. In addition, a *T*-test indicated that age of participants did not differ significantly between both samples, *t*(461) = 2.98, *p* = 0.085. *T*-tests (see Table 1) revealed that social capital and satisfaction with life exhibited no significant differences between samples, whereas Instagram use differed significantly between both samples. Active and passive Instagram use was assessed higher in sample 1 compared to sample 2. Thus, it was concluded that both samples differed in terms of sample characteristics and Instagram use. Because of these sample differences the hypotheses were tested separately in each sample.

**Table 1:** Descriptive Statistics of Both Samples and t-Tests of Sample Differences

Scale	Sample 1		Sample 2		*t(461)*	*p*	Cohen’s *d*
	*M*	*SD*	*M*	*SD*			
IG-a	2.68	0.67	2.26	0.65	6.43	.001	0.64
IG-p	3.54	0.71	3.32	0.57	3.17	.002	0.36
SC-bo	3.09	1.12	3.06	1.10	0.27	.789	0.03
SC-br	3.25	0.87	3.12	0.78	1.58	.114	0.16
SWLS	5.20	1.19	5.16	1.11	0.36	.723	0.04

*M* = mean, *SD* = standard deviation, *t* = t-value, *dfs*_1_ = 141, *dfs*_2_ = 318; IG-a = Instagram active, IG-p = Instagram passive, SC-bo = Social Capital bonding, SC-br = Social Capital bridging, SWLS = Satisfaction with Life Scale

### Intercorrelations

As indicated by the analysis of skewness and kurtosis (Appendix Table 5), almost all variables both in sample 1 and 2 were approximately normal distributed. Only passive Instagram use in sample 2 was not normally distributed. Consequently, instead of Pearson’s correlation coefficient, the non-parametric correlation coefficient by Spearman was applied for this scale. The correlations are summarised in Table 2. Cohen (1988) denoted effect sizes from 𝑟𝑥𝑦 = .10 as small effects, from 𝑟𝑥𝑦 = .30 as moderate effects, and from 𝑟𝑥𝑦 = .50 as strong effects.

**Table 2:** Intercorrelations of the Used Scales for Both Samples

Scale	Sample	IG-a	IG-p	SC-bo	SC-br
IG-a	1	–			
	2	–			
IG-p	1	.531***	–		
	2	.462^a^***	–		
SC-bo	1	.220**	.317***	–	
	2	.156**	.224^a^***	–	
SC-br	1	.434***	.480***	.403***	–
	2	.429***	.427^a^***	283***	–
SWLS	1	−.125	.102	.295***	.072
	2	−.060	.063^a^	.132*	.074

*dfs*_1_ = 141, *dfs*_2_ = 318, ^a^ = rank correlation according to Spearman; 1 = Sample 1, 2 = Sample 2; IG-a = Instagram active, IG-p = Instagram passive, SC-bo = Social Capital bonding, SC-br = Social Capital bridging, SWLS = Satisfaction with Life Scale. *p < .05, **p < .01, ***p < .001

Both domains of Instagram use were significantly positively correlated with both domains of social capital. Quite strong effects were revealed for the associations between both domains of Instagram use and bridging social capital and small to moderate effects were registered for the association between Instagram use and bonding social capital. The highest positive correlation was found between passive Instagram use and bridging social capital (explaining 18.23–23.04% of variance). Therefore, *H1a-d* were confirmed.

Furthermore, both Instagram use and social capital were conceptualized as two-dimensional constructs. For both samples, active and passive Instagram use were significantly positively correlated among each other (explaining 21.34% and 28.19% of variance) representing strong effects. Both domains of social capital were also significantly positively correlated (explaining 8.01% and 16.24% of variance) representing moderate effects. For Instagram use neither the passive nor the active domain revealed a significant correlation with satisfaction with life. Therefore, *H2a-b* were not confirmed.

Additionally, as hypothesised, only the bonding and not the bridging domain of social capital displayed a significantly positive correlation with satisfaction with life (explaining 1,74–8,70% of variance) representing a small effect for sample 1 and a moderate effect for sample 2. Therefore, *H3* was confirmed.

### Mediation

**Fig. 1:**
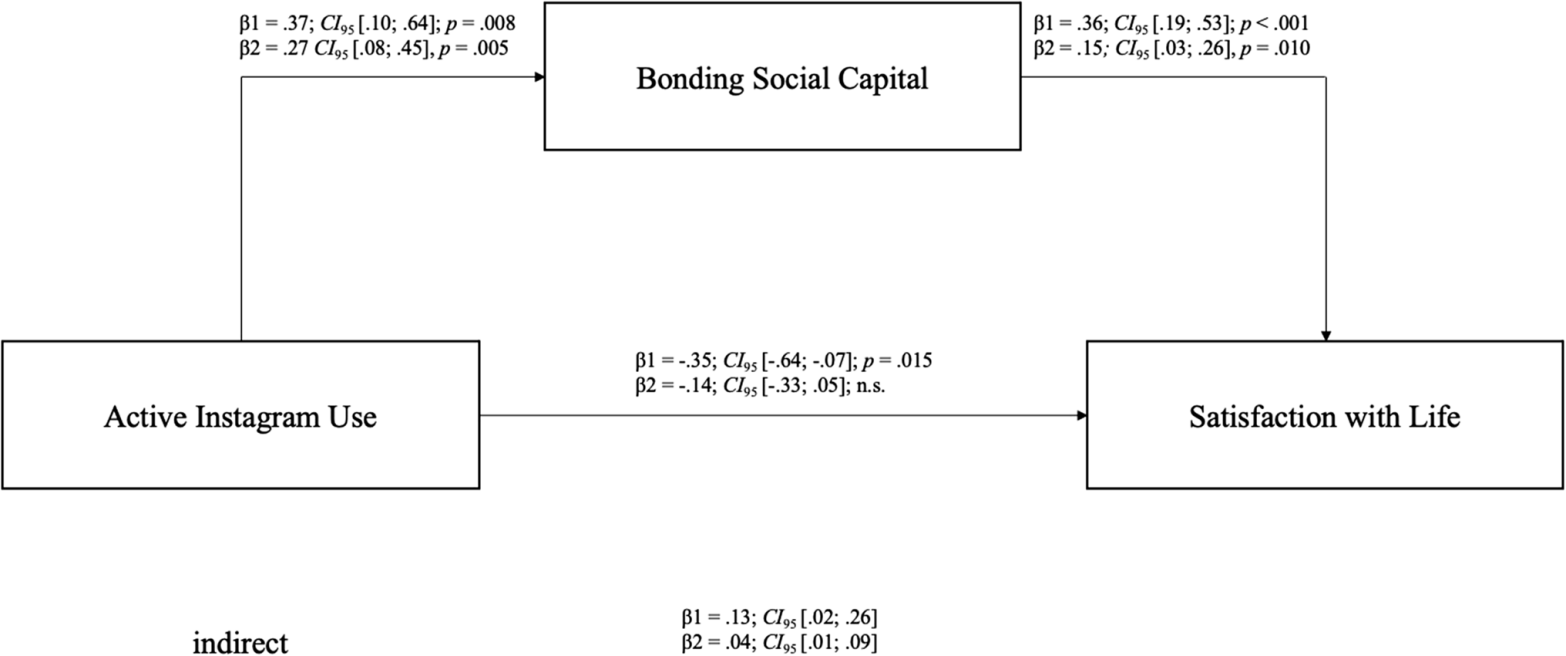
Mediation Model for Both Samples Predicting Satisfaction with Life with Active Instagram Use. *Note.* β = regression coefficient, *dfs*_1_ = 1141, *dfs*_2_ = 1318, *m* = 10,000; *F1*,141 = 2.24, *p* = .137, *R*^2^ = .13, *F1*,318 = 1.14, *p* = .286, *R*^2^ = .06; confidence intervals in brackets

Based on *H4*, mediation analyses were conducted using model 4 of the Script PROCESS 3.5 by Hayes (2018, p. 149). Model 4 is generally used when conducting a simple mediation analysis with additional options that can be specified for running the analysis (Field, 2018). This procedure is based on OLS-regression analysis as well as additional bootstrapping analyses (sample size *r* = 10,000). The necessary assumptions for mediation analysis are independence, linearity, normal distribution, homoscedasticity, and independence of the residuals (Hayes, 2018, p. 80). These conditions were tested and confirmed. The mediation model is summarised in Fig. 1 for both samples. For sample 1 the indirect and the direct path was significant because the confidence interval did not include zero (indirect: β = .13 *CI*_95_[.02; .26], direct: β = −.35 *CI*_95_[−.64; −.07], *p* = .015). Note that the mediation was only partial because the association between active Instagram use and satisfaction with life was still significant after controlling for the mediator (Hayes, 2018). The total effect was not significant with β = −.22 *CI*_95_[−.51; .07], *p* = .137, but the values showed that the negative direct effect from active Instagram use to satisfaction with life was reduced by the mediator. 12.50% of variance in satisfaction with life could be explained by the model with bonding social capital as a mediator, *F1*,141 = 2.24, *p* = .137, *R*^2^ = .13 for sample 1. For sample 2, only the indirect effect was significant, β = .04 *CI*_95_[.01; .09], whereas the direct and total effect was not significant (direct: β = −.14 *CI*_95_[−.33; .05], *p* = .143; total: β = −.10 *CI*_95_[−.29; .09], *p* = .286). 6.00% of variance could be explained by the mediation model for sample 2, *F1*,318 = 1.14, *p* = .286, *R*^2^ = .06. Prior studies stated that the effect of a mediation is primarily described by the indirect effect. They suggest that it is sufficient to interpret the indirect effect alone (Field, 2018; Hayes, 2018; Rucker et al., 2011; Zhao et al., 2010). Therefore, *H4* was basically confirmed. The results indicated the occurrence of a positive effect of bonding social capital on the association between active Instagram use and satisfaction with life.^1^

### Replicability

A test of excessive significance based on the mediation model of both samples was conducted. A success rate of 0.71 was revealed. Therefore, 71.00% of the hypotheses could be confirmed, achieving a median observed power of 0.71. The inflation rate was 0.002 and the R-Index 0.704 indicating that the findings could be replicated in 70% of follow-up studies. Thus, the data does not seem to be biased and findings turn out to be sufficiently generalizable.

## Discussion

This study introduces a novel measure of Instagram use based on behavioural reports. Note that behavioural reports are better indicators of behaviour than attitude measures (Fishbein & Ajzen, 1975) They are closer to behaviour representing direct behaviour indicators. Therefore, behavioural reports are in general considered as good measures of behaviour (Kaiser et al., 2010). The IAQ is based on frequency assessments. The newly developed Instagram Activity Questionnaire consists of 30 items. Based on factor-analytic results two different domains of Instagram use were delineated: *active* and *passive*.

The two-factor structure of the IAQ is consistent with recent research dividing SNS use into active and passive domains (cf., Burke et al., 2010; Krasnova et al., 2013; Verduyn et al., 2015). Note that a recent review, which examined 27 studies on active and passive social media use, points to underlying methodological limitations such as heterogenous content (cf., Valkenburg et al., 2021). Further research is needed to clarify these issues.

Both subscales of the IAQ achieved good internal consistency. They correlated positively, but the common variance was not higher than 28%. Furthermore, support for construct and convergent validity of the IAQ was obtained. This is, to our knowledge, the first proposal of a measure specifically for Instagram activity based on behavioural reports. The advantage of such a valid and reliable measure is obvious. Note that the factor-analytic results are based on a sample of more than 450 respondents meaning that the factor structure of the 30 items is likely to be quite stable. For additional validation of the factor model of the IAQ, replication studies are needed. We hope that future studies may benefit from the IAQ which we consider as a reliable and valid behavioural report reflecting - with high accuracy - Instagram behaviour of the respondents.

In accordance with previous research (Putnam, 2000), social capital was operationalized as a two-dimensional measurement instrument. The two-dimensional approach seems to be successful in this domain. Specifically, both subscales exhibited good internal consistencies. In addition, the intercorrelations of the subscales were in a moderate range indicating that the subscales tap a common underlying dimension of social capital. At the same time, they capture different facets of social capital (bridging vs. bonding).

The aim of this study was to examine how Instagram use, social capital, and satisfaction with life are associated with each other. In general, hypothesis tests were replicated in both samples indicating high generalizability of results across samples. In *H1* it was hypothesized that both domains of Instagram use are positively correlated with mobilization of social capital (be it bridging or bonding social capital; *H1a-d)*. Results of this investigation, which are consistent with prior research (cf., Ellison et al., 2007; Phua et al., 2017; Steinfield et al., 2008), confirm these assumptions. The highest correlations were found between both domains of Instagram use and bridging social capital (up to 23% common variance), representing moderate effects. However, the correlations between bonding social capital and Instagram use were also significantly positive (up to 10% of common variance), representing small effects. Thus, SNSs users seem to be rather positively interacting and communicating with others on Instagram (cf., Shane-Simpson et al., 2018; Waterloo et al., 2018) building up bonding social capital and especially bridging social capital.

It was hypothesized in *H2* that active Instagram use is positively correlated with satisfaction with life, whereas passive Instagram use is negatively correlated with satisfaction with life. However, across both samples no significant associations of Instagram use and satisfaction with life were observed. Therefore, *H2* is rejected based on the results in both samples. Note that an alternative hypothesis is summarized in *H4* (see below).

*H3* refers to the association between bonding social capital and satisfaction with life. Specifically, *H3* was confirmed because bonding social capital and satisfaction with life were positively associated. No corresponding significant association emerged for bridging social capital. These results were consistent for both samples. Only bonding social capital was systematically related to satisfaction with life. An explanation for the missing association with bridging social capital refers to the way participants evaluate their satisfaction with life. Judgements on satisfaction with life are strongly based on the specific and current context conditions (Schwarz & Strack, 1999). Therefore, confirming prior research on satisfaction with life (cf., Burke et al., 2010), weak ties summarised under bridging social capital may not be as important as strong ties summarised under bonding social capital.

The mediation model proposed that the association of active Instagram use and satisfaction with life is mediated by bonding social capital. It was scrutinized and confirmed in both samples. To simplify matters, the antecedent condition is denoted with X, the outcome with Y and the mediator with M (cf., Hayes, 2018). Specifically, positive direct paths were revealed for Instagram use and bonding social capital (X → M) as well as for bonding social capital and satisfaction with life (M → Y). The mediation analysis of the link between X and Y depends essentially on the confirmation of the systematic connection between X and M and between M and Y (Hayes, 2018). Because these links were significant (supporting *H1a* and *H3*) the preconditions for conducting a mediation analysis between X and Y via M were fulfilled. Furthermore, a small negative direct association was found between active Instagram use and satisfaction with life. This link between X and Y was not significant disconfirming *H2*. But as Hayes (2018, p. 88) in accordance with Bollen (1989) points out: “On the surface, it seems that the existence of an association between X and Y would be a reasonable precondition of trying to explain the underlying effect of X on Y. But there has been a growing recognition over the last few years that such thinking it misguided.” In correspondence with this clarification, the indirect effects are significant for both samples and reveal that a significant mediation effect via bonding social capital occured. This mediation model explains up to 12.50% of total variance. Active Instagram use was positively associated with bonding social capital which in turn was positively related to satisfaction with life. Therefore, *H4* was twice confirmed. In summary, the confirmation of *H1a*, *H3,* and *H4* replicated in two samples represents impressive evidence for the mediation model. Bonding social capital mediated the association between active Instagram use and satisfaction with life. But because of the correlational design of the study, it is not possible to infer causation from the confirmation of the mediation model.

Because the inferences are based on a correlational design, the results are also compatible with an alternative framework which starts with satisfaction with life as a predictor of active Instagram use. Although such a framework is conceivable, it is not likely to be viable. Although the correlational data allows no causal inference, their interpretation should correspond with plausibility. Based on the social capital theory, it is implausible to assume that satisfaction with life leads to active Instagram use. Furthermore, such an assumption is also not congruent with research indicating that social relationships enhance satisfaction with life and not vice versa. Remarkably, social relationships are considered to be the most important source of satisfaction with life (Berscheid & Reis, 1998). Empirical evidence from a study in Chicago indicated that involvement in a social network of persons who are trustworthy, responsive and supportive contributed to the accumulation of social capital, which was associated with lower mortality rates in the neighborhood (Lochner et al., 2003). In addition, cross-cultural research across 31 nations (including USA, Japan, and Germany) indicated that close relationships like family and friends are positively related to life satisfaction (Diener & Diener, 1996). More specifically, persons who live in steady relationships express more happiness than singles, widows, or divorced (Myers, 1999). Furthermore, meta-analytic results summarizing longitudinal data indicated that the occurrence of marriage increased satisfaction with life in accordance with a *honeymoon effect* (Luhmann et al., 2012). In summary, the availability of social connections and the occurrence of social capital gains seem to foster satisfaction with life and well-being (cf., Ellison & Vitak, 2015; Rohmann & Bierhoff, 2016).

Furthermore, satisfaction with life is derived from thinking about one’s own life situation (Diener et al., 1985). In correspondence with the proposed mediation model, it is likely that the current life situation is influenced by active Instagram use and its accompanying effects on accumulating bonding social capital.

In addition, replicability analyses showed that, theoretically, the same effects occur in around 70% of replication studies. Overall, these results indicate that bonding social capital mediated the association between active SNS use and satisfaction with life. Therefore, bonding social capital may be an important factor for a better understanding of SNS use and the consequences it has on individuals’ well-being. Therefore, bonding social capital should be taken into account when explaining the association between active Instagram use and satisfaction with life. The confirmation of *H4* is intriguing, because it points to the positive resources, which are implied by bonding social capital in the context of active Instagram use. It might have positive effects on your well-being, but only mediated via bonding social capital, which stands for the presence of close social relationships on the Instagram platform.

### Limitations and Future Research

This investigation has several limitations. The first point is that males and older users of SNSs are underrepresented in this investigation. Up to 85% of the participants were female and nearly 86% under the age of 30 which makes the results less transferable to males and older persons. However, most people who use Instagram are quite young, so distorting effects regarding age of respondents with respect to Instagram use might be small. Nevertheless, further studies should try to replicate the results within a larger and more balanced sample. Another point regarding the sample is that many participants were psychology students (up to 53%) which may also have restricted the generalizability of the results.

Another limitation this study encountered was the public atmosphere in which the collection of the data occurred for sample 2. From the middle of March 2020 to the end of April 2020 many restrictions on personal and social life were enacted due to the coronavirus (Covid-19) pandemic. Although both samples do not significantly differ with respect to social capital and satisfaction with life, they differed significantly for both domains of Instagram use with somewhat higher values of Instagram use for sample 1 than for sample 2. These differences must not necessarily be due to the changes of daily life for everyone in the shadow of the pandemic, because it would rather be expected that values for Instagram use increase during lockdown (Statista, 2020). Nevertheless, it might be useful to replicate this investigation when daily life parameters return to levels before the pandemic.

It must also be mentioned that this study is based on a correlational design. The hypotheses were delineated from theoretical considerations and previous research. They are compatible with a causal interpretation although the correlational design excludes causal inferences from the results. Significant mediation does not imply true mediation but only that the data fits with the proposed mediation model (Fiedler et al., 2011; Hayes, 2018). Future experimental studies could more convincingly prove that the proposed causal direction of the mediation model is valid.

It is common to classify effect sizes in psychology according to Cohen (1988), but recent literature provides evidence that small effect sizes represent the norm because of high variability in the genetics and – therefore – in the behavior of human beings (Götz et al., 2021). Large effects are rarely found (Funder & Ozer, 2019). However, this recent evidence about the magnitude of effect sizes strengthens the assumption that the findings of our study are relevant.

The variance explained by the mediation model is relatively small for both samples (i.e., sample 1 12.50%, sample 2 6.00%) and the results suggest only partial mediation (at least in sample 1). Therefore, it is likely that further variables mediate the association between active Instagram use and satisfaction with life which might be investigated in future studies. Furthermore, future investigations might focus on which specific aspects of SNSs lead to accumulation of bonding social capital and how people who increase their well-being employ SNSs. It is also very interesting to examine the hypotheses of this investigation for other SNSs (e.g., Snapchat, Facebook, Twitter, LinkedIn etc.). Instagram is, in contrast to other SNSs, heavily picture-based and elicits predominantly positive emotions (Waterloo et al., 2018). These features might facilitate the accumulation of bonding social capital among Instagram users. Whether the important mediating role of bonding social capital as a mediator between active Instagram use and satisfaction with life is also confirmed for other platforms like Facebook remains an open question. One speculation in this context is that social relations of Instagram users include primarily strong ties, whereas other SNSs like Facebook attract more social exchange in weak-ties networks. This is an interesting hypothesis for future research. Therefore, social capital theory might improve the development of a theoretical framework of the consequences of use of SNSs which incorporates differences in features of several SNSs (cf., Hellemans et al., 2020). The concept of social capital seems to be the key to the elaboration of the psychological consequences of distinct communication features which are offered by different SNSs. For example, on Instagram users might primarily accumulate bonding social capital, whereas on Facebook they might gain more bridging social capital (cf., Ellison et al., 2007).

In conclusion, this investigation on social media revealed associations between Instagram use, social capital, and satisfaction with life which partly replicate previous study results and partly enter new territories of theory and research. Specifically, with respect to entering new research territories, a new measure of Instagram use was delineated. This IAQ proved to be reliable and valid. Because the IAQ is based on behavioral reports derived from frequency assessments, its employment goes beyond subjective ratings of preferences offering more objective data. The availability of such a behavioral measure, which distinguishes active from passive Instagram use, is likely to facilitate future research on Instagram use considerably.

In addition, new territory was entered by the proposition that bonding social capital serves as a mediator between active Instagram use and satisfaction with life. The confirmation of the mediation model established a link between Instagram use and satisfaction with life which fits well into social capital theory. In addition, the confirmation of the respective mediation model in two samples provides a possible resolution of contradictory results obtained in prior research. Furthermore, although the hypothesis of direct links between Instagram use and satisfaction with life was disconfirmed by the results, the inclusion of bonding social capital as a mediator variable pointed out that an indirect link between active Instagram use and satisfaction with life via bonding social capital must be considered as an alternative. Finally, the hypothesis tests were replicated consistently in two independent samples indicating that the results exhibit a considerable level of empirical generalizability.

## Data Availability

The datasets generated and analyzed during the current study are available in the OSF repository, https://osf.io/yw298/?view_only=8a287c68db1d4cfcb7973fca456b3a2f.

## Appendix 1

**Table 3:** Item Structure and Factor Loadings of the Two-Factor Instagram-Activity Scale

Scale	Item	Factor loading
Active	Es gibt Familienfotos von mir auf Instagram. [On Instagram are family pictures of me.]	0.463
	Auf meinen Fotos ziehe ich Grimassen (z.B. lustig, süß, sexy). [I am making faces on my pictures (funny, cute, sexy, etc.)]	0.450
	Ich bearbeite meine Profilbilder, bevor ich sie einstelle. [I edit my profile pictures before uploading them.]	0.595
	Ich bearbeite meine Fotos, bevor ich sie einstelle. [I edit my pictures before uploading them.	0.608
	Ich verwende Filter für meine Fotos. [I use filters for my pictures.]	0.566
	Meine Fotos zeigen mich in Aktion (z.B. beim Sport oder bei der Arbeit). [My pictures show me in action (e.g., playing sport or working).]	0.418
	Auf meinen Fotos bin hauptsächlich ich zu sehen. [On my pictures you can see mainly me.]	0.426
	Ich stelle Fotos ein, auf denen ich wie ein Model posiere. [I post pictures on which I pose like a model.]	0.587
	Ich stelle Fotos ein. [I post pictures.]	0.637
	Ich stelle Videos ein. [I post videos.]	0.513
	Ich verwende aktiv die Story-Funktion. [I am actively using the story-function.]	0.548
	Ich verwende die Story-Highlight Funktion. [I am using the story-highlights function]	0.534
	Ich plane gezielt, wann ich ein Foto poste. [I plan specifically when I post a picture.]	0.572
	Ich lösche ältere Fotos. [I delete older pictures.]	0.506
	Ich lösche Fotos, die nicht beliebt sind. [I delete pictures which are not popular.]	0.559
	Ich entferne Hashtags. [I delete hashtags.]	0.548
	Ich schaue regelmäßig, wie viele Leute meine Seite abonniert haben. [I look regularly how many people subscribe to my site.]	0.544
	Ich achte darauf, dass mein Profil einen guten Gesamteindruck ergibt. [I take care that my profile has a good overall appearance.]	0.594
	Ich überlege genau, welchen Text ich als Fotobeschreibung verwende. [I think carefully which text I use as description of my pictures.]	0.533
Passive	Ich schaue mir die Kurzbio anderer Nutzer an. [I look at the bio of others.]	0.541
	Ich schaue mir die Fotos anderer Nutzer an. [I look at pictures of others.]	0.717
	Ich lese mir die Kommentare zu den Fotos anderer Nutzer durch. [I read comments on pictures of others.]	0.558
	Ich lese mir die Fotobeschreibungen anderer Nutzer durch. [I look at the description of others pictures.]	0.696
	Ich schaue mir die Hashtags anderer Nutzer an. [I look at hashtags of others.]	0.370
	Ich schaue mir die Stories anderer Nutzer an. [I look at stories of others.]	0.678
	Ich schaue mir die Story-Highlights anderer Nutzer an. [I watch the story-highlights of others.]	0.486
	Ich schaue mir die Videos anderer Nutzer an. [I watch videos of others.]	0.596
	Ich like Fotos anderer Nutzer. [I like pictures of others.]	0.609
	Ich lese Privatnachrichten, die andere Nutzer mir senden. [I read private messages that other users send to me.]	0.558
	Ich sende Privatnachrichten an andere Nutzer. [I send private messages to other users.]	0.527

## Appendix 2

**Table 4:** Intercorrelations of Instagram Use and the Facebook-Activity Scale for Sample 1

	IG-a	IG-p	FB-W	FB-A	FB-I
IG-a	–				
IG-p	.531***	–			
FB-W	.240**	.205*	–		
FB-A	.449***	.220*	.563***	–	
FB-I	.235**	.277**	.448***	.548***	–

*dfs* = 141; IG-a = Instagram active, IG-p = Instagram passive, FB-W = Facebook watching; FB-A = Facebook acting; FB-I = Facebook impressing

**p* < .05, ***p* < .01, ****p* < .001

## Appendix 3

**Table 5:** Skew and Kurtosis statistics of Instagram scales, social capital scales and Satisfaction with Life Scale

Scale	Sample 1		Sample 2	
	Skew	Kurtosis	Skew	Kurtosis
IG-a	0.078	−0.058	0.037	−0.508
IG-p	−0.438	0.734	−0.760	1.902
SC-bo	−0.333	−0.962	−0.309	−0.878
SC-br	−0.569	0.281	−0.453	0.047
SWLS	−0.488	−0.053	−0.652	0.524

*N1* = 143, *N2* = 320. IG-a = Instagram active, IG-p = Instagram passive, SC-bo = Social Capital bonding, SC-br = Social Capital bridging, SWLS = Satisfaction with Life Scale

## Footnotes

1 Mediation analyses have been calculated controlling for gender and student status. However, no significant covariations occurred (all *p*s > .05)
